# Iron Protein Succinylate in the Management of Iron Deficiency Anemia: A Comparative Study with Ferrous Sulphate at Low and High Therapeutic Doses

**DOI:** 10.3390/nu13030968

**Published:** 2021-03-17

**Authors:** Katia Urso, Javier Leal Martínez-Bujanda, Jaime Moscoso del Prado

**Affiliations:** ITF Research Pharma S.L.U., San Rafael 3, 28108 Madrid, Spain; jleal@itfsp.com

**Keywords:** iron protein succinylate, hepcidin, anemia, IDA, ferrous sulphate, iron deposition, Ferplex, oral treatment

## Abstract

Oral iron supplementation constitutes the first line treatment for iron deficiency anemia (IDA), with daily doses between 80 mg and 200 mg of elemental iron. Ferrous salts, such as ferrous sulphate (FeSO_4_), while efficacious, frequently give rise to gastrointestinal side effects. In the present paper we attempted to directly compare the efficacy of an alternative to the FeSO_4_ formulation, which presents a better tolerability profile, iron protein succinylate (Ferplex^®^). In a diet-induced anemia model, rats were treated by oral gavage with vehicle, FeSO_4_, or Ferplex^®^ at a human-dose equivalent of 80 mg and 200 mg of elemental iron. We evaluated the change in anemia-related hematological and biochemical parameters, conducting a histological examination of the intestine at sacrifice. Results indicate that both types of iron supplementation are equally effective in the treatment of IDA, restoring hemoglobin, hematocrit, erythrocytes, free iron and transferrin levels in 15 days, with no statistical differences between treated groups and control. The impact of anemia on body weight was also attenuated following treatment with both iron supplements. Thrombocyte and reticulocyte levels, altered by the anemic condition, returned to homeostasis after 15 days of either FeSO_4_ or Ferplex^®^ treatment. Importantly, the lower and higher doses of iron were equally effective, thus supporting the current school of thought which states that lower therapeutic doses are sufficient for management of IDA. In addition, the study shows for the first time that oral treatment with Ferplex^®^ does not increase serum hepcidin. Finally, Ferplex^®^ induced minimal iron depositions in the intestinal tissue compared to FeSO_4_.

## 1. Introduction

Iron deficiency is the principal cause of anemia [1] and can be produced by malnutrition, blood loss (gastrointestinal or menstruation bleeding) or by chronic diseases, including inflammatory bowel disease, chronic kidney disease or cancer [2]. IDA causes weakness, fatigue [3], and in severe cases can jeopardize mental and motor development in children and increase the risk of child and maternal mortality [4].

The first-line treatment of iron deficiency anemia involves oral iron administration with recommended doses ranging between 80–200 mg of iron/day [2,5]. Ferrous sulphate (FeSO_4_) is the gold standard among iron treatments for anemia. However, it frequently produces adverse effects in the gastrointestinal tract such as nausea, vomiting, constipation and diarrhea [6,7]. The average duration of treatment is 3–6 months, but common side effects result in a non-adherence to therapy in up to 50% of patients, thus preventing effective correction of iron deficiency anemia. Unabsorbed iron is generally deemed to be the cause of these gastrointestinal side effects [7].

As the use of FeSO_4_ to treat IDA is compromised by poor compliance as well as the aforementioned side effects, efforts have focused upon developing alternative formulations including either ferrous (+II, bivalent) and ferric (+III, trivalent) iron sources. While other ferrous salts such as ferrous fumarate and ferrous gluconate are comparable with FeSO_4_ in terms of side effects, the ferric formulation in the form of iron protein succinylate (Ferplex^®^) is known to exhibit an improved safety profile both by reducing the incidence of adverse events (including gastrointestinal adverse events) and by reducing the severity and duration thereof [8,9,10].

Oral ferric compounds are generally known to have lower bioavailability than ferrous compounds, due to their poor solubility in neutral to alkaline environments [8]. In the particular case of iron protein succinylate, an improved pH-dependent mechanism of absorption gives rise to greater bioavailability of iron when compared with classic ferric compounds.

Ferplex^®^ is a protein complex containing 5% ferric iron bound to succinylated casein. The formulation was designed to keep the iron bound to the protein matrix when the complex is in acid pH environments (stomach), limiting the occurrence of gastrointestinal adverse events, and releasing the ferric iron ions when in neutral to alkaline pH (within the duodenum and the proximal jejunum). Ferric iron displays enhanced solubility when presented in complexes with organic ligands such as Ferplex^®^. These complexes prevent precipitation of large polynuclear hydroxides at neutral pH values, typical of other ferric compounds, thus ensuring iron availability in the duodenum and the proximal jejunum. Moreover, the succinic acid contained in the complex further increases intestinal iron absorption by up to 30% [11,12,13].

Early pharmacological studies have shown that administration of iron protein succinylate elevates serum iron levels in a slightly delayed but more sustained manner over time when compared with FeSO_4_. This guarantees a gradual absorption while achieving similar iron concentrations; moreover, both iron protein succinylate and FeSO_4_ achieved higher serum iron concentrations than those provided by other ferric compounds [14].

A recent systematic review of clinical and observational studies referring to the last 30 years demonstrated that ferric iron complex of succinylated protein achieved superior efficacy and tolerability in comparison with other ferric salts, along with a much lower rate of adverse effects than that of FeSO_4_ and other ferrous preparations. In addition, treatment duration with iron protein succinylate was on average 15.5% shorter (49 versus 58 days) as compared to that of the ferrous comparators [9].

Iron absorption is regulated by the hepatic hormone hepcidin, which controls the release of iron to plasma from absorptive sites and the release of iron from stores [15]. Hepcidin transcription is triggered by high plasmatic levels of iron and by systemic inflammation. In turn, the induced hepcidin limits further absorption of iron, resulting in a negative feedback mechanism. Likewise, when hepcidin levels are low, dietary iron is readily absorbed from the enterocytes [15,16]. Oral administration of FeSO_4_ is known to induce hepcidin transcription 24 h after administration, thus limiting the bioavailability of administered iron [17,18]. The hepcidin increase is dose-dependent and produces a 35–45% decrease in the fractional iron absorption [19]. In order to address this issue, recent studies have suggested that, to increase iron absorption and prevent the negative impact of increased hepcidin levels, lower doses and/or an intermittent dosing scheme could be implemented [19,20].

Based on recent evidence and considering the known capability of Ferplex^®^ to produce more gradual iron absorption [14], we hypothesized that treatment with the lower clinical dose of Ferplex^®^ (equivalent to 80 mg elementary iron/day) would be as effective as higher doses in restoring the physiological levels of anemia-related parameters. Mechanistically, combining the gradual absorption of Ferplex^®^ with a low dose regime, likely bypassing hepcidin induction, might constitute an efficient treatment.

Consequently, the present research attempts to compare the efficacy of the two standard iron supplements, iron protein-succinylate (Ferplex^®^) and FeSO_4_, administered in two therapeutic doses: a low dose (human equivalent of 80 mg) and a high dose (human equivalent of 200 mg), in the treatment of IDA in a rat model. We evaluated the analytical and physiological parameters associated with anemia during a 14-day treatment period, in line with previous studies [21,22]. Additionally, we investigated the potential effect of Ferplex^®^ upon hepcidin induction as compared with FeSO_4_, a treatment known to induce high hepcidin expression.

## 2. Materials and Methods

### 2.1. Animals

The study involved male Spraque-Dawley weaning rats at the BioAdvice facility, Lyshøjvej 21, Ølstykke, Denmark under license No. 2015-15-0201-00540 and was approved by the ethical committee of the Animal Experiments Inspectorate of Denmark. The male Sprangue-Dawley rats were purchased from Janvier Labs, France. On arrival, the rats presented an average body weight of 65.2 g (56.6–72.6 Min-Max) and an age of 3–4 weeks. Animals were maintained in a 12-h light/dark cycle under a controlled environment at a temperature of 20–23 °C and a relative humidity of 30%–70%. Upon arrival, rats were randomly allocated into 7 groups of 8 animals (4 animals per cage) as described in Table 1.

On the day of arrival, the animals were offered *ad libitum* either standard chow (a complete pelleted standard rodent diet “Altromin 1324”, Fe-content of 192.51 mg/kg diet, from Brogaarden, Denmark) or an Fe-restricted diet (a specially formulated iron-restricted rodent diet “Altromin C 1038”, Fe-content of 5.16 mg/kg diet, from Brogaarden, Denmark).

An additional group was included to monitor and establish the onset of the anemic conditions. A mean hemoglobin level below 9 g/dl was considered to constitute a marker of anemia [23] and treatment with test compounds was initiated. All 8 animals in the monitoring group reached an anemic hemoglobin concentration by day 19.

Treatment was initiated after 19 days on a given Diet, and this stage is considered as Day 0 of treatment. Treatment was applied once daily for 14 days by oral gavage with Ferplex^®^, FeSO_4_ or a vehicle, as specified in Table 1. Ferplex^®^ is already manufactured as a ready-to-use solution, FeSO_4_ was resuspended in drinking water. Final concentration of both solutions was 2.67 mg/mL of elementary iron. During the treatment period, the rats were weighed once daily prior to administration of test compounds to ensure accurate dose calculation. Dosing was performed daily at 12.00 h (± 30 min).

Human to rat dose was converted as previously described [24]. The equivalent of human iron daily dose of 80 mg and 200 mg was 7.1 mg/kg and 17.7 mg/kg, respectively. The Ferplex^®^ and FeSO_4_ treatments were provided by Italfarmaco S.A. and by Dr. Paul Lohmann GmbH, respectively.

Rats were monitored for pain or distress and euthanized as specified by humane endpoint criteria according to the Animal Experiments Inspectorate of Denmark (endpoints include clinical, behavioral or pathophysiological signs of pain, distress or fear, such as activity, posture, aggression, response to handling, respiration rate, dehydration, …).

### 2.2. Procedures

**Figure 1:**
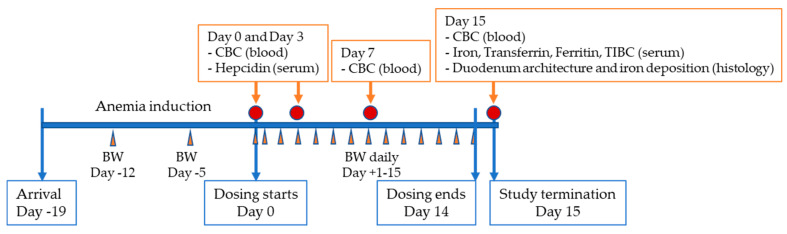
Study timeline. CBC: Complete blood count included measurement of hemoglobin, mean corpuscular volume, hematocrit and hemogram; BW: Body weight; TIBC: Total iron binding capacity.

Figure 1 shows the timeline for procedures, which is described in detail below.

Clinical observation and body weight: All visible signs of ill health and behavioral changes (activity, posture, fur, eyes, respiratory rates, etc.) were recorded daily during the study. Any deviation from normal was recorded with respect to time of onset, duration, and intensity. During the treatment period, the rats were weighed once daily and before administration of the test compounds to ensure accurate dose calculation.

Hematological test: Blood samples (400 μL) were collected from the sublingual vein in Lithium-Heparin coated tubes (Microvette^®^, Sarstedt, art. No. 20.1345.100), on days 0, 3 and 7 or were extracted by cardiac puncture (2 mL) at study termination on day 15. The blood samples were used for a complete blood count (CBC) including hemoglobin (Hb), red blood cells (RBC), hematocrit (HCT) and mean corpuscular volume (MCV). The analysis was performed with a fully automatic hematology analyzer (ADVIA 2120i Siemens).

Serum samples and analysis: Additional blood samples were collected from the sublingual vein (250 μL) on days 0 and 3 in serum tubes with a clotting activator (Microvette^®^, Clotting Activator/Serum) and left for 1 h to clot. After clotting, the samples were centrifuged for 15 min in a refrigerated centrifuge (set to maintain 4 °C) at 2500× *g*. The resultant serum was transferred to clear polypropylene tubes (Micronic tubes) and frozen immediately over dry ice and placed in a freezer at −80 °C. Moreover, TIBC and Iron were analyzed by means of a fully automatic enzyme-based assay (Advia 1800 Siemens). Hepcidin content was analyzed by ELISA ((hepcidin: LS-F24076, LSBio, Seattle, WA, USA). The serum obtained from part of the blood taken at termination by cardiac puncture was used to measure transferrin and ferritin by means of ELISA (transferrin: ab137993, ferritin: ab157732 both from Abcam, Cambridge, UK) and TIBC and Iron were measured via a fully automatic enzyme-based assay (Advia 1800 Siemens). Transferrin saturation was calculated indirectly based on the TIBC and iron values with the following formula: Transferrin saturation = iron (µmol/L)/TIBC (µmol/L) × 100 [25].

Histological analysis: For histology, tissue samples measuring approximately 2 cm were excised from the duodenum post-mortem, starting distally from the pyloric opening; they were fixed in 10% formalin, dehydrated, and embedded in paraffin. To maximize the area of the tissue section, the duodenal tissue was longitudinally orientated in the paraffin blocks. Sections measuring 5-μm were cut longitudinally at the midline of the paraffin-embedded duodenal samples. Subsequently, one section was stained with haematoxylin/eosin and another section with a Prussian blue iron staining kit (Abcam). The stained sections were examined blinded with a light microscope with a ×20 lens and an ocular ×10 providing a magnification of ×200. Villus and crypt height were determined using ImagePro plus 9.2 software (Media Cybernetics, Rockville, MD, USA). Mucosa height was determined as the sum of the villus and crypt heights. The non-luminal iron staining was scored by two blinded independent observers experienced in gastrointestinal histopathology; they employed a semiquantitative scale from 0–4; 0 indicated absence of iron staining in the enterocytes and 4 represented the relative maximum iron staining observed therein.

Data analysis: Graph representation and statistical analysis were performed with the use of GraphPad Prism 9.0.0 software (La Jolla, CA, USA). Results are expressed as mean ± SEM. The Data available for each parameter were tested for normality distribution at every time point examined (Shapiro Wilk test). Many of the parameters did not provide a normal distribution. For this reason, in addition to the limited sample size of the groups, the most conservative non-parametric test was selected to analyze the data. The Mann–Whitney test was used for two-group analysis (iron deposition histology) and the Kruskal–Wallis test, followed by Dunnett’s test, was employed for the comparison of multiple groups, as indicated in each figure legend. In the intermediate time points, where indicated, the treatment groups were compared with each other to evaluate potential differences between iron source or dose. At study termination all treatment groups were compared with each other and with the control group to confirm restoration of physiological levels. A *p* value <0.05 was considered to be significant.

## 3. Results

### 3.1. Treatment with Ferplex^®^ and FeSO_4_ Reverses Diet-Induced Anemia

**Figure 2:**
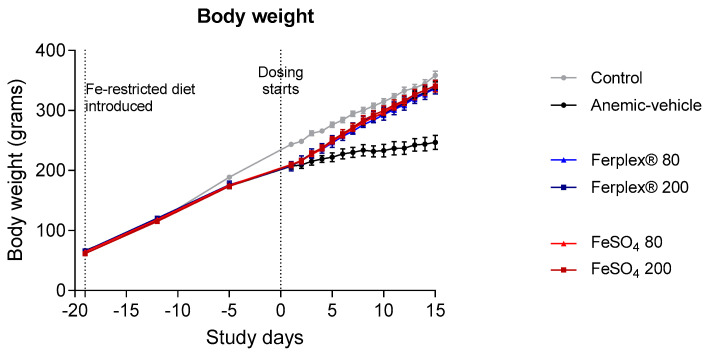
Body weight monitoring during anemia induction and treatment period. Weights were monitored daily from day 0 to day 14, when the study was terminated. Data are presented as mean ± SEM. No significant difference was found between treatment groups (Ferplex^®^ versus FeSO_4_) or between the 80 vs. 200 mg doses. No statistical difference was found between treatment groups and control at study termination. Data were analyzed by means of a Kruskal-Wallis test and with Dunnett’s multiple comparison test. N = 6 (anemic-vehicle and Ferplex 200 at day 15), 7 (FeSO_4_ 80 and 200), 8 (control and Ferplex^®^ 80).

Anemia was induced by administration of an Fe-restricted diet in male Spraque-Dawley rats. After 19 days, when hemoglobin concentration was below 9 g/dL, rats on the Fe-restricted diet were treated for 14 days either with vehicle, Ferplex^®^ or FeSO_4_ (both of them at doses equivalent to human doses of 80 mg and 200 mg of elemental iron) by oral gavage, mimicking the clinical procedure of a once daily oral treatment. Body weight was monitored daily during the treatment phase until termination of the study. As expected, IDA affected growth, as the anemic rats presented a significantly lower body weight compared with those kept on a standard chow diet (*p* < 0.01 at day 1), as demonstrated previously [25]. Treatment with either Ferplex^®^ or FeSO_4_ rapidly restored normal bodyweight (*p* < 0.05 on day 15, comparing treatment groups versus anemic-vehicle groups; not significant between treatments and control). The effects of the low and high doses were similar (Figure 2).

**Figure 3:**
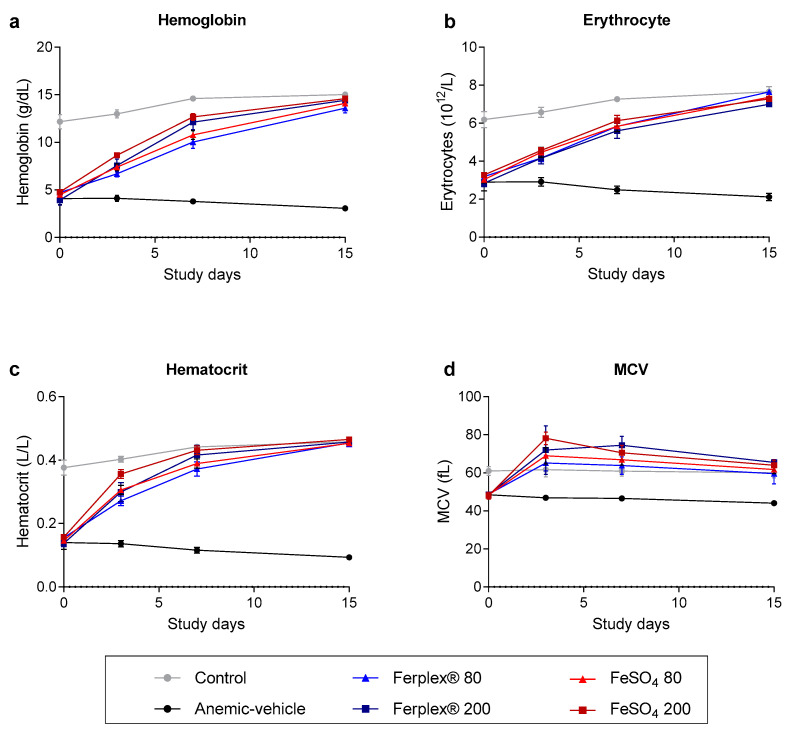
Complete blood count monitored during the treatment period. (**a**) Hemoglobin, (**b**) erythrocyte count (**c**) hematocrit and (**d**) mean corpuscular volume (MCV) measured in blood samples on days 0, 3, and 7 and at study termination on day 15. Data are presented as mean ± SEM. No significant difference was observed between treatment groups (Ferplex^®^ versus FeSO_4_) and doses. Neither was any significant difference detected between treatment groups at any time point. At termination there was no significant difference between treatment groups and control. Data were analyzed by means of a Kruskal-Wallis test followed by Dunnett’s multiple comparison test. N = 5–8.

We then analyzed blood samples on days 0, 3, 7 and at study termination on day 15. As expected, the concentration of hemoglobin, hematocrit and erythrocyte count were found to be lower in the rats on the Fe-restricted diet compared to rats on the standard diet. At the start of the treatment (day 0) the rats in the anemic-vehicle group were seen to be extremely anemic, with a hemoglobin concentration of approximately 4.6 g/dL; no significant difference was observed between anemic groups. Iron supplementation with either Ferplex^®^ or FeSO_4_ completely restored physiological levels of hemoglobin, hematocrit and erythrocyte count after 14 days of treatment. (Figure 3). No significant difference was found between the two doses (80 mg versus 200 mg) in either Ferplex^®^ or FeSO_4_ nor between the two iron sources at any time point. At study termination, no significant difference was observed between any of the iron treatments and the standard chow group.

Additionally, MCV showed an increase in all groups receiving either Ferplex^®^ or FeSO_4_ at the beginning of the treatment period as compared with the control group. However, at study termination the MCV of all treatment groups returned to physiological levels (Figure 3d). Moreover, in this case, no significant difference was detected between the type of iron supplementation and doses at any time point. Likewise, at study termination no significant difference was found between the groups receiving iron supplementation and the control chow group.

**Figure 4:**
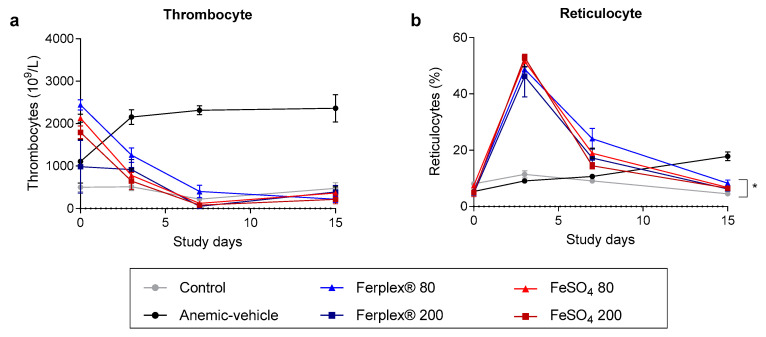
Cell populations affected by anemia during the treatment period. (**a**) The number of thrombocytes and (**b**) Reticulocyte percentage was recorded in blood samples on days 0, 3, and 7 and at study termination on day 15. Data are presented as mean ± SEM. There was no significant difference between treatment groups at any time point. * *p* < 0.05 Ferplex^®^ 80 mg as compared with the control group on day 15 with a Kruskal-Wallis test followed by Dunnett’s multiple comparison test. N = 5–8.

Anemia resulting from iron deficiency leads to secondary thrombocytosis, a condition characterized by excessive thrombocyte production [26]. As expected, thrombocyte count increased drastically in the anemic-vehicle group in relation to that of the control group. Treatment with either Ferplex^®^ or FeSO_4_ rapidly restored physiological levels of thrombocytes and no difference was observed between the 80 mg and 200 mg doses (Figure 4a).

Iron supplementation is known to increase the percentage of total red blood cells (reticulocyte percentage) at the beginning of the treatment period, which is indicative of an increase in erythropoiesis [27,28]. As the erythrocyte count approached normal levels half-way through the treatment period, the percentage of reticulocytes gradually returned to physiological levels. No difference was detected at study termination between treatments/doses and the control chow group, with the exception of Ferplex 80 mg. However, although statistically significant, the difference can be considered to be clinically and physiologically irrelevant (Figure 4b).

**Figure 5:**
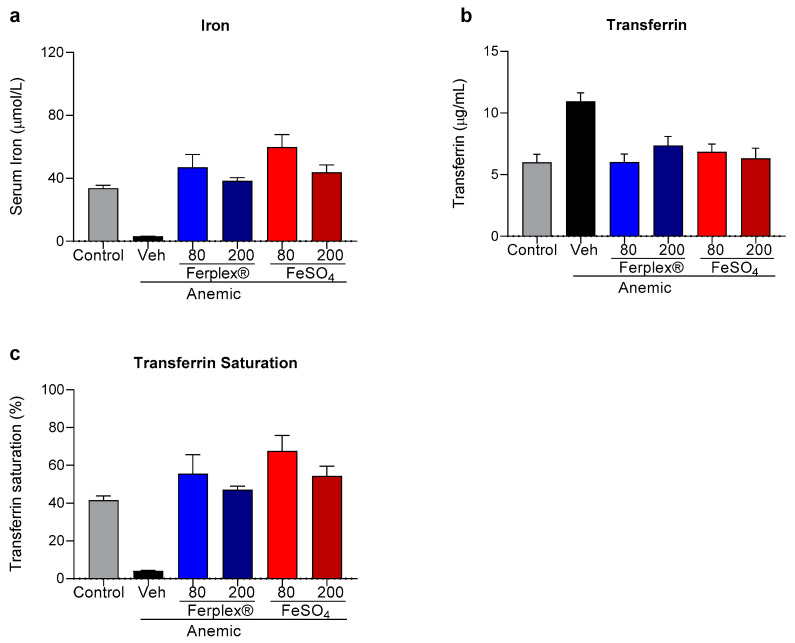
Iron and transferrin analysis at study termination. Concentration of iron (**a**) and transferrin (**b**) recorded in serum at study termination. (**c**) Percentage of transferrin saturated. Data are presented as mean ± SEM. At termination there was no significant difference between treatment groups and control. Data were analyzed with a Kruskal-Wallis test followed by Dunnett’s multiple comparison test. N = 6 to 8 (as indicated in the Table 2). Veh = vehicle.

The biochemical markers of anemia were analyzed in the serum samples collected at study termination. As expected, iron content was significantly lower in the anemic-vehicle rats, in which the deficiency was compensated by an increase in transferrin concentration (Figure 5a,b). Both treatments, Ferplex^®^ and FeSO_4_, completely restored physiological concentrations of both parameters. The anemia also induced a drop in saturated transferrin concentration, which was recovered similarly by Ferplex^®^ and FeSO_4_ (Figure 5c). In these three biochemical parameters, concentrations were restored to similar levels regardless of the dose at study termination.

Surprisingly, Ferritin and TIBC values at study termination maintained physiological levels in all groups, including the anemic-vehicle group. The lack of difference is likely due to the short duration of the anemic condition (Table 2).

### 3.2. The Effect of High-Dose Ferplex^®^ and FeSO_4_ on Intestinal Mucosa

**Figure 6:**
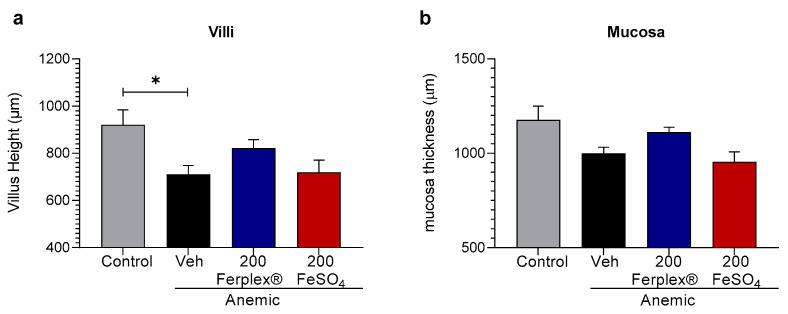
Histological HE analysis of villi (**a**) and mucosa (**b**) height in duodenal samples. Data are presented as individual points and mean ± SEM. * *p* < 0.05 with the Kruskal-Wallis test followed by Dunnett’s multiple comparison test. N = 6 (anemic-vehicle); 8 (control); 7 (treatment groups). Veh = vehicle.

Long-term and/or high dose treatment with iron supplements has been frequently shown to cause damage in the intestinal mucosa [29]. Considering the short duration of the treatment (only 14 days), we explored the possible deleterious effect of iron supplementation on the architecture of the duodenal tissue only in the high-dose groups. As the results indicate, neither of the treatments produced major histological alterations. Only a mild effect of the anemia itself was observed. Indeed, the rats on an iron-restricted diet presented shortened intestinal villi compared to the rats on the standard diet. This finding confirms previously published clinical studies which show shortening of the villi in iron-deficient anemic children [30,31]. Herein, no significant effect of the Ferplex^®^ or FeSO_4_ treatments on the height of the villi was observed, although a tendency towards the recovery of physiological villus height can be observed in the Ferplex^®^ treated group (Figure 6a). Similar trends can be observed in the thickness of the mucosa (Figure 6b). The lack of significant histological effects is likely due to the relatively short period of the treatment.

**Figure 7:**
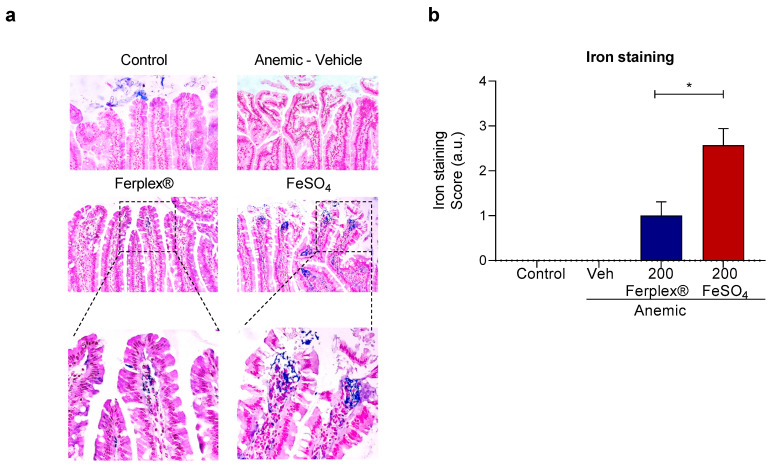
Histological analysis of iron deposition. (**a**) Representative image of Prussian blue staining of the duodenal sample, showing localization of blue iron staining in the lamina propria at 200x magnification. (**b**) Quantification of iron deposition as a score of non-luminal iron staining. * *p* < 0.05 with the Mann-Whitney test. N = 6 (anemic-vehicle); 8 (control); 7 (treatment groups). Veh = vehicle.

Sections of the intestinal samples were stained with Prussian blue staining for iron deposition and scored with a predetermined scoring system (0–4) of intracellular staining. As expected, no iron deposition was detected in the anemic-vehicle rats. The rats on standard chow occasionally showed positive blue staining in the intestinal lumen. The samples from rats treated with Ferplex^®^ or FeSO_4_ exhibited different degrees of iron staining localized in the lamina propria (Figure 7). The iron staining score was significantly lower in the rats treated with Ferplex^®^ when compared with the rats treated with FeSO_4_.

### 3.3. Iron Supplementation and Serum Hepcidin Induction

**Figure 8:**
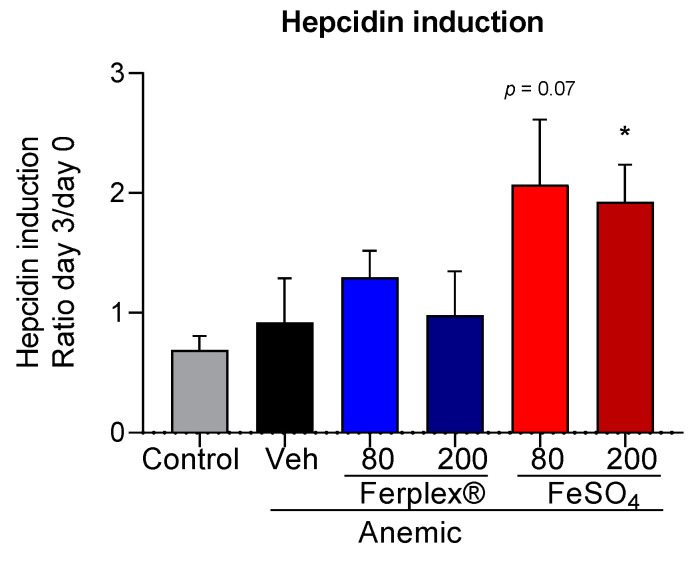
Hepcidin induction calculated as the ratio between the concentration of serum hepcidin measured in serum on day 3 and on day 0 of the treatment via an ELISA assay. * *p* < 0.05, as compared with the control group by means of a Kruskal-Wallis test, followed by Dunnett’s multiple comparison test. N = 5 (FeSO_4_ 200); 7 (control); 8 (Ferplex^®^ 80); 6 (remaining groups). Veh = vehicle.

Hepcidin induction has been described to occur at an early stage of the treatment with iron supplements. In the clinical setting, an increase in serum hepcidin concentration was observed in as little as 24 h after FeSO_4_ administration [19]. Consequently, we recorded the plasmatic content of serum hepcidin on day 0 (basal level before treatment) and at the earliest time point (day 3) of the treatment in all groups. At baseline (day 0, before administration of the first oral dose) serum hepcidin concentration in the anemic groups (pooled, 1477 ± 148 pg/mL) was statistically lower than control group (2053 ± 136 pg/mL) (*p* < 0.05, Mann-Whitney test). To evaluate the effect of the treatments, we evaluated the change in hormone concentration (ratio day 3/day 0). In accordance with previous studies, the FeSO_4_ treatment raised serum hepcidin concentration (approximately 2-fold), displaying a significant difference between the FeSO_4_ 200 mg treatment and the control group. The FeSO_4_ 80 mg group, although not statistically significant (*p* = 0.07), showed a similar trend. On the contrary, in the groups treated with Ferplex^®^, serum hepcidin concentration remained stable (fold induction approximately 1) at this early time point, similar to what was observed in the control and anemic-vehicle groups. No difference was seen to exist between the two doses of Ferplex^®^ (Figure 8).

## 4. Discussion

Ferric salts have shown an improved safety profile as compared with the gold standard, FeSO_4_, and other ferrous salts [11]. However, due to the low solubility of most ferric compounds in the intestine, these preparations are generally absorbed less effectively than ferrous compounds [8]. In contrast, iron protein succinylate is highly soluble in neutral to alkaline pH [11,12], and has been observed to be as effective as classic ferrous preparations such as FeSO_4_, while providing much better tolerability results. The rate of adverse events of iron protein succinylate is over three times lower than that of FeSO_4_ [9].

Our study is the first to compare different doses of two iron sources, iron protein succinylate (Ferplex^®^) and FeSO_4_, for treatment of anemia^®^. In agreement with previously published literature [9,10,32], herein Ferplex^®^ has demonstrated comparable efficacy to FeSO_4_ for reversal of iron deficiency.

In line with recent studies, we have demonstrated that a once daily iron dose of 80 mg is equally effective as a 200 mg dose with regard to reversing the induced anemia. Moretti et al. showed that daily FeSO_4_ supplementation in iron-depleted young women is maximized by means of elemental iron doses of 40–80 mg once per day when compared with higher doses of 120–160 mg [19]. Furthermore, a follow up study by Stoffel et al. also suggests that higher doses potentially increase side effects due to the excess of unabsorbed iron remaining in the gastrointestinal tract [18]. This study supports these findings, but more research is needed to explore doses even lower than 80 mg per day.

The possibility of reducing conventional dosing has been suggested following the discovery of the important role played by the principal regulator of the iron metabolism, hepcidin. It is known that, while low concentrations of hepcidin are necessary for absorption of orally administered iron, an increase in hepcidin occurs 24 h after FeSO_4_ administration, limiting the bioavailability of administered iron [17,18,33,34]. Moreover, the increase in hepcidin is dose-dependent [30] and might account for the low absorption rate of oral iron supplements. In agreement with the literature, in our study treatment with FeSO_4_ caused an increase in hepcidin concentration.

However, we found no difference in serum hepcidin induction between low and high doses of FeSO_4_. Moretti et al. found that 80 mg of FeSO_4_ is sufficient to induce hepcidin, but they also revealed that higher doses of FeSO_4_ induced higher concentrations of hepcidin after one single day of iron supplementation [19]. In our study, we evaluated hepcidin concentration after three consecutive days of iron supplementation. We hypothesize that hepcidin levels rise immediately after the first FeSO_4_ administration in a dose-dependent manner, but that repeated administrations of lower therapeutic doses of FeSO_4_ may achieve a hepcidin induction similar to that of higher therapeutic doses, at least after three days of iron supplementation. Further studies are needed to explore the dose-response effect of FeSO_4_ on hepcidin induction under chronic conditions.

Another open question involves the kinetics of hepcidin expression after day 3 and how this relates to changes in serum iron levels. A future line of investigation should focus more on the specific impact of the iron sources over time on these two parameters in parallel. We interpreted our results as indicating that the hepcidin induced by FeSO_4_ supplementation further reduces iron absorption and maintains homeostatic levels of iron. In the case of Ferplex, the rate or mechanism of absorption results in reaching homeostatic levels of plasmatic iron without the need of the hepcidin intervention to limit further absorption. However, we cannot discard the possible participation of other pathways, independent from hepcidin, that might be triggered by Ferplex and could impact the concentration of free iron available in the blood.

Little is known of the impact of different types of iron supplementation upon hepcidin induction. Herein, we have shown for the first time that administration of iron via Ferplex^®^ does not induce serum hepcidin. However, further studies are needed to explore the mechanism of action, possibly related to the kinetics of absorption, underlying this effect.

Ferritin concentration was measured at study termination but, surprisingly, we found no difference between groups, especially between control and anemic-vehicle. It is, however, interesting that all treatment groups show mean ferritin values marginally above the anemic group. Nonetheless, the groups are small and, as there is a relatively large variability (especially in the control group) it is difficult to understand whether this is reflecting a real difference. We can only speculate that the lack of difference might be due to the short duration of the anemia which may not have been sufficient to significantly impact iron storage, that is the main function of ferritin.

Additionally, at histological level, we observed that in the rats treated with high doses of FeSO_4_, the iron absorbed by the enterocytes is not fully exported, with a fraction remaining inside the cells. This trapped iron could pose a safety risk in relation to the gastrointestinal mucosa. In contrast, the iron staining score was significantly lower in the rats treated with Ferplex^®^ 200 mg. The increase in hepcidin concentration after oral administration of iron supplement is known to be able to block the export of iron from enterocytes to plasma [35,36,37]. Consequently, one might also expect the hepcidin induced after FeSO_4_ supplementation to be responsible for the iron retained inside the enterocytes. We therefore postulate that the fact that Ferplex^®^ did not induce hepcidin in these experimental settings might be related to the lesser accumulation of iron inside the enterocytes, since lower concentrations of serum hepcidin imply a higher export of intracellular iron.

The principal limitation of our study refers to the number of observations we collected per group. Nonetheless, despite the limited number of animals employed in the study, most of the data provided thereby are sufficiently robust to support our main conclusions and to highlight the need for further corroboration of this exploratory research,

Another limitation involves the length of our study. The main goal thereof was to investigate potential differences in efficacy between the two iron sources and, based on the literature, two weeks is sufficient to observe a full recovery from anemia [21,22]. Therefore, a short study and a low dose were specifically selected as a strategy aimed at establishing potential differences between the two regimes. The short duration of the study, however, possibly means that we have relinquished valuable information referring to the potential local effect of iron supplementation on the intestinal mucosa. This aspect will be further investigated in a long-term study.

One additional weakness of this study is that it was conducted only in male rats to limit variability, since it has previously been suggested that sex-dependent differences exist in homeostatic iron metabolism, including basal concentration of hemoglobin, ferritin and hepcidin [38]. Although we have no reason to suspect that males and females use different mechanisms to develop IDA or to respond to iron supplementation, this factor should be considered in future clinical and preclinical studies.

In general terms, our study in vivo suggests the fact that Ferplex^®^ has comparable efficacy to FeSO_4_ in the treatment of IDA. Results support Ferplex^®^ as a valuable alternative to FeSO_4_, due to the fact that the former exhibits equal efficacy and improved tolerability. Moreover, we have demonstrated that doses of 80 mg of elemental iron are as efficacious as doses of 200 mg for reversing IDA, thus reiterating the recent tendency to question the current dosing scheme in an attempt to improve the management of IDA.

Efficacious and well-tolerated formulations such as Ferplex^®^, administered in low therapeutic doses, may help to optimize the efficacy and safety results of oral IDA treatment. There is a need for randomized clinical trials to confirm the therapeutic equivalence of lower and higher iron doses in IDA management.

## Figures and Tables

**Table 1:** Description of diet and treatment of respective groups.

Group Name	Diet	Treatment
Control	Standard chow	Vehicle (water)
Anemic-vehicle	Fe-restricted diet	Vehicle (water)
Monitor group	Fe-restricted diet	None
Ferplex^®^ 80 mg	Fe-restricted diet	Elemental Fe 7.1 mg/kg
Ferplex^®^ 200 mg	Fe-restricted diet	Elemental Fe 17.1 mg/kg
FeSO_4_ 80 mg	Fe-restricted diet	Elemental Fe 7.1 mg/kg
FeSO_4_ 200 mg	Fe-restricted diet	Elemental Fe 17.1 mg/kg

**Table 2:** Concentration of other biochemical markers on day 15.

Group Name (n)	Ferritin ng/mL (Mean ± SEM)	TIBC µmol/L (Mean ± SEM)
Control (8)	863 ± 131	81.6 ± 1.3
Anemic-vehicle (6)	894 ± 83	77.2 ± 3.4
Ferplex^®^ 80 mg (8)	1048 ± 90	83.2 ± 1.9
Ferplex^®^ 200 mg (6)	1483 ± 156	81.3 ± 1.5
FeSO_4_ 80 mg (7)	1330 ± 171	87.9 ± 2.2
FeSO_4_ 200 mg (7)	1274 ± 175	80.2 ± 1.6

TIBC: Total iron binding capacity.
